# Pharmacological effects on 35% CO_2_ panic induction: A meta-analysis

**DOI:** 10.1177/02698811251378756

**Published:** 2025-10-29

**Authors:** Jette H. de Vos, Alissa Haj Yahya, Wolfgang Viechtbauer, David E. J. Linden, Koen R. J. Schruers, Nicole K. Leibold

**Affiliations:** 1 Department of Psychiatry and Neuropsychology, Mental Health and Neuroscience Research Institute (MHeNs), Maastricht University, the Netherlands; 2 Department of Health Psychology, University of Leuven, Belgium

**Keywords:** panic attacks, carbon dioxide, psychopharmacology, emotions

## Abstract

**Background:**

A brief inhalation of 35% CO_2_ triggers subjective fear and physiological responses occurring during naturally occurring panic attacks (PAs). This CO_2_ model enables to study effects of pharmacological interventions on experimental panic provocation and examine the biological mechanisms involved in PAs.

**Aims:**

To provide a quantification of the effects of pharmacological interventions on the response to CO_2_ inhalation, which was still lacking despite decades of research and numerous studies having addressed these effects.

**Methods:**

A systematic search was performed to identify peer-reviewed papers reporting effects of pharmacological interventions to the 35% CO_2_ inhalation. Multilevel meta-analyses were performed to quantify the effects of such interventions on self-reported anxiety and PA symptoms.

**Results:**

Thirty-six studies, containing data of 980 participants (both panic disorder patients and healthy individuals), were included. Several studies reported effects of multiple pharmacological interventions, resulting in 48 effect sizes for the meta-analysis of the effects on anxiety and 34 for the effects on PA symptoms. Significant decreases in induced anxiety (−.55 (95% confidence interval (CI): −.81 to −.29), *p* < 0.0001) and PA symptoms (−0.31 (95%CI: −.51 to −.11), *p* = 0.0026) were seen after pharmacological interventions aimed at symptom reduction. Induced anxiety was significantly decreased (−.81 (95%CI: −1.13 to −.48), *p* < 0.0001) after pharmacological interventions that enhanced the serotonergic system.

**Conclusions:**

This meta-analysis supports the notion that specific drugs can reduce the sensitivity to 35% CO_2_ challenge, supporting a role for this procedure as experimental model to investigate panic pharmacology.

## Introduction

Every fifth person in the general population experiences a panic attack (PA) at least once in their lives (Kessler et al., 2006). PAs are characterized by a burst of intense symptoms such as dizziness, palpitations, chest pain, a feeling of choking, and/or overwhelming fear. PAs are the hallmark of panic disorder (PD; Cougle et al., 2010), which is characterized by pervasive anxiety for the occurrence of more attacks. Moreover, PAs can be used as a specifier of all mental disorders in the Diagnostic and Statistical Manual of Mental Disorders-5 (2013; Craske et al., 2010). The marked impairments in quality of life and the accompanying high health care costs (Batelaan et al., 2007) drive the need to understand the psychophysiological mechanisms of PAs and to develop treatments that address them.

A brief inhalation of 35% CO_2_ has been found to trigger the fear and physiological responses occurring during naturally occurring PAs (Schruers et al., 2004). Over years of research, this inhalation has proven to be a well-validated model of real-life PAs (Leibold et al., 2015, 2016). This PA-like response can be provoked in both PD patients and healthy volunteers in a non-invasive, relatively easy, and safe manner (Leibold et al., 2013, 2015; Nardi et al., 2006; Perna et al., 2004; Schruers et al., 2011). Moreover, 35% CO_2_-induced PAs have also been reported in other psychiatric disorders in which PAs are common, such as post-traumatic stress disorder (Kellner et al., 2018). First-degree relatives of patients with PD (Perna et al., 1995) show a higher sensitivity to the inhalation than healthy controls (HCs), suggesting a genetic component to CO_2_ sensitivity levels. Enhanced CO_2_ sensitivity has been identified as intermediate phenotype for PD and to some extent for certain anxiety disorders (Gottesman and Gould, 2003; Leibold et al., 2015; Meyer-Lindenberg and Weinberger, 2006). Specifically, patients with social anxiety disorder have a sensitivity lower than that of PD patients but higher than that of healthy individuals (Schutters et al., 2012), as only part of social anxiety symptomatology is reproduced.

One opportunity the CO_2_ model offers is to study the effects of pharmacological interventions on anxiety/PA symptoms induced by experimental provocation. This also creates the possibility of studying the biological mechanisms involved in PAs. With this aim, studies have been performed with a wide variety of manipulations, studying both inhibitors and facilitators of the response to the CO_2_ inhalation. For example, manipulations that increase or decrease the availability of serotonin have been administered prior to the CO_2_ inhalation (Colasanti et al., 2011; Schruers and Griez, 2004), which aided in understanding the role of the serotonergic system in PAs.

Another major opportunity provided by the CO_2_ model is its predictive value for the clinical effects of medication, based on which it can be used as a screening tool to determine the therapeutic potential of new compounds. For example, the magnitude of the response to a CO_2_ inhalation after 1 week of serotonin transporter inhibitor (selective serotonin reuptake inhibitor (SSRI)) treatment was predictive of the clinical outcome after 1 month of SSRI treatment (Perna et al., 2002). Moreover, benzodiazepines (GABAergic modulators), which are effective in the treatment of PD, also inhibit CO_2_-induced PAs (Nardi et al., 2000). Conversely, drugs without clinical efficacy do not affect the response to the CO_2_ inhalation either (Papadopoulos et al., 2010).

Despite decades of research and hence a large number of studies having addressed the effects of pharmacological interventions on the response to the CO_2_ inhalation, a comprehensive quantification of the effect of such pharmacological interventions is still lacking. A quantification of this could further establish the value of the CO_2_ inhalation as an experimental model of PAs and as a screening tool for pharmacological interventions for these attacks. As mentioned above, several studies show analogies between drugs that work on the CO_2_ response as well as on real PAs, but a meta-analytic quantification of this could strengthen the notion of the CO_2_ inhalation as a screening tool. To address this gap, we undertook a systematic search, followed by a meta-analysis. The primary aim of this meta-analysis was to assess the effects of pharmacological interventions intended to inhibit the CO_2_-induced panic response. Exploratory, we also assessed the effects of pharmacological interventions intended to enhance the panic response. The secondary aim was to determine whether pharmacological interventions targeting different biological systems have a different effect (i.e. inhibitory or facilitatory effect) on the response to the CO_2_ inhalation.

## Materials and methods

The results of this meta-analysis are reported following the guidelines outlined by the Preferred Reporting Items for Systematic Reviews and Meta-analysis (PRISMA) statement (Moher et al., 2009). The protocol was registered in the PROSPERO database (CRD42023412146) prior to conducting the meta-analysis.

### Search strategy and study selection

Relevant studies were identified by conducting a search of existing literature in two databases until July 2025 without restriction on the starting date: PubMed and Web of Science Core Collection. The search strategy was as follows: (“Panic disorder” OR “Panic”) AND (“carbon dioxide” OR “CO2” OR “CO 2”); the full search terms used in PubMed and Web of Science can be found in the Supplement. After removing duplicates, the titles and abstracts of the papers were first screened for eligibility. If the abstract was not available, the paper was included in the full-text screening. Removal of duplicates and screening decisions were tracked in Rayyan (Ouzzani et al., 2016). Subsequently, full texts of the selected studies were screened for eligibility. Both screening steps were performed by two independent reviewers blinded to each other’s decision (authors J.V. and A.H.Y.). After unblinding, conflicts in eligibility decisions were resolved by re-checking and further discussing source papers between reviewers until consensus was reached. If needed, a third investigator (author K.S.) was consulted about the inclusion decision.

Studies had to meet the following criteria to be included: (1) inclusion of patients with PD (presence of comorbidities allowed) or healthy volunteers, (2) use of a pharmacological intervention, (3) use of a 35% CO_2_ inhalation, with data available from a CO_2_ inhalation under the influence of the pharmacological intervention and a control inhalation (under the influence of a placebo, or if not available, a CO_2_ inhalation performed at baseline, i.e. prior to the pharmacological intervention), (4) use of original data, (5) availability of predefined self-report outcome data (see below which self-report data were included), and (6) published in English in a peer-reviewed journal. Posters, conference abstracts, letter to editors, and commentaries were excluded.

### Data collection

Data extraction was performed independently by two researchers (J.V. and A.H.Y.), and mismatches were resolved by discussion until consensus was reached. The following data were extracted from the included articles: (1) sample size, (2) mean age, (3) sex distribution, (4) sample characteristics: PD patients with or without comorbidities or healthy volunteers, (5) CO_2_ procedure used: single or double vital capacity breath of 35% CO_2_, (6) aim/hypothesis of the study: inhibit or increase response to the inhalation, (7) characteristics of the pharmacological intervention: name of the manipulation, used dose, and for how long the medication was taken, and (8) names, means, and standard deviations of the reported outcome measures.

### Analysis

For the analysis, the self-reported outcome measures were classified either as a measure of anxiety (e.g. a visual analogue scale of anxiety or an anxiety rating on a Likert scale) or a questionnaire assessing PA symptoms (e.g. the panic symptom list or the panic symptom inventory). As information about the correlation between the two outcomes was not reported in the studies, precluding the possibility of a multivariate meta-analysis, all analyses were conducted separately for those two outcome variables.

Several scenarios of designs and reported data were seen in the included papers. The majority of studies used a pure within-subjects design that measured the outcome once before and once after the CO_2_ inhalation under a control condition and after administration of the pharmacological intervention (treatment). For studies of this type, we computed the standardized mean change (SMC) using raw score standardization with $d=\frac{\left({\bar{x}}_{\mathrm{post}}^{T}-{\bar{x}}_{\mathrm{pre}}^{T}\right)-\left({\bar{x}}_{\mathrm{post}}^{C}-{\bar{x}}_{\mathrm{pre}}^{C}\right)}{{\mathrm{SD}}_{\mathrm{post}}^{\mathrm{pool}}}$ , where ${\bar{x}}_{\mathrm{post}}^{T}$ and ${\bar{x}}_{\mathrm{pre}}^{T}$ are the post- and pre-inhalation means for the treatment condition, ${\bar{x}}_{\mathrm{post}}^{C}$ and ${\bar{x}}_{\mathrm{pre}}^{C}$ are the corresponding means for the control condition, and ${\mathrm{SD}}_{\mathrm{post}}^{\mathrm{pool}}$ is the pooled post-inhalation standard deviation. This way, any potential differences prior to the inhalation and any differences in the overall response to the CO_2_ inhalation were taken into account. In a few cases, the pre-inhalation means were not assessed or reported, in which case we computed the effect size as. On the other hand, for studies using a pretest–posttest control group design, we computed the difference in the SMC between the conditions with $d=\frac{\left({\bar{x}}_{\mathrm{post}}^{T}-{\bar{x}}_{\mathrm{pre}}^{T}\right)}{{\mathrm{SD}}_{\mathrm{post}}^{T}}-\frac{\left({\bar{x}}_{\mathrm{post}}^{C}-{\bar{x}}_{\mathrm{pre}}^{C}\right)}{{\mathrm{SD}}_{\mathrm{post}}^{C}}$ , where ${\mathrm{SD}}_{\mathrm{post}}^{T}$ and ${\mathrm{SD}}_{\mathrm{post}}^{C}$ are the post-inhalation standard deviations of the respective groups. In case the pre-inhalation means were not assessed or reported, we computed the regular standardized mean difference with $d=\frac{\left({\bar{x}}_{\mathrm{post}}^{T}-{\bar{x}}_{\mathrm{post}}^{C}\right)}{{\mathrm{SD}}_{\mathrm{post}}^{\mathrm{pool}}}$ . By always using the (pooled) raw score standard deviation of the post-inhalation measurements in the denominator, we ensured that the different effect size types were numerically comparable (Morris and DeShon, 2002). In all cases, the appropriate bias correction (i.e. to correct for the slight positive bias in small sample sizes) was applied to the effect sizes.

When computing the SMC of studies with a within-subjects design, a correlation of 0.60 (anxiety measures) or 0.65 (PA measures) was assumed between the change scores of repeated CO_2_ inhalations. These correlations were based on in-house published data of repeated CO_2_ inhalations (de Vos et al., 2024). When only post-inhalation data were reported in the included studies with a within-subjects design, a correlation of 0.65 (anxiety measures) or 0.60 (PA measures) was assumed between the post-inhalation scores of repeated CO_2_ inhalations. When computing the SMC of studies with a between-subjects design, a correlation of 0.40 (anxiety measures) or 0.30 (PA measures) was assumed between the pre- and post-measurements. These correlations were based on in-house unpublished and published (Leibold et al., 2013) data of CO_2_ inhalations.

Some studies included in the meta-analysis allowed calculating an effect size for different pharmacological interventions. For studies where the control group or condition was used repeatedly to calculate these effect sizes, this induces a correlation of approximately 0.50 for the sampling errors, which we also assumed for the subsequent calculation. Based on this assumption and the calculated sampling variances of the *d*-values, we constructed an approximate variance-covariance matrix of the estimates, which was then used, together with the *d*-values, as input to the meta-analysis models. In analyses where some studies provided multiple estimates, we used a multilevel random-effects model (Konstantopoulos, 2011), adding random effects at the study and effect size levels. Otherwise, a standard random-effects model was used, with random effects only at the effect size level (but accounting for dependency in the sampling errors via the variance-covariance matrix of the estimates where necessary). Based on a particular model, we report the estimated pooled (average) effect with 95% confidence interval (CI), the results of the *Q*-test for heterogeneity, the $I^{2}$ statistic (at the study and effect size level for multilevel models), and the 95% prediction interval (PI).

To answer our first research question, namely what the effects are in studies that aimed/hypothesized to inhibit the CO_2_ response with a pharmacological intervention, we fitted the model in this subset of studies. To test our second research question, namely, whether pharmacological interventions targeting different biological systems differ in their effect on the response to the CO_2_ inhalation, we fitted a meta-regression model with pharmacological target as moderator. Effect sizes were classified based on the main target of the related pharmacological intervention. In order to make a meaningful comparison between the different biological systems, given the number of effect sizes per system, three groups of pharmacological targets were made (defined as serotonergic, GABAergic, or another target), allowing to address the differential effects between serotonergic and GABAergic compounds, and between serotonergic or GABAergic compounds compared to other pharmacological targets.

The same analyses were repeated in the subset of studies that aimed/hypothesized to enhance the CO_2_ response. We decided to run separate models on the studies that aimed/hypothesized to inhibit versus enhance the CO_2_ response (as described above), as a direct comparison between those two sets of studies was deemed to be uninformative due to the considerably different nature of those studies. However, within the studies targeting the serotonergic system, we fitted another meta-regression model to explore the differential effects of interventions that enhanced or inhibited the effect of serotonin signalling in the brain, either by modulating the levels of serotonin (e.g. via the reuptake of serotonin or by modulating the levels of serotonin precursors) or for example by blocking a serotonin receptor. This model will highlight the role of the serotonergic system in the CO_2_ response.

As a sensitivity analysis and to account for potential misspecification of the models used for the analyses, we compared the results from the fitted models with those obtained when using cluster-robust inference methods (Pustejovsky and Tipton, 2022). This led to no change in conclusions and hence we do not report these results below. Additional sensitivity analyses were performed by using standardized residuals and Cook’s distances to identify potential outlying and/or influential studies, which were then subsequently excluded from an analysis. In order to assess the risk of bias due to “small-study effects,” funnel plots were created (Sterne et al., 2005), and regression tests for asymmetry were performed on the plot. For these plots and tests, we approximated the standard errors of the d-values with $\sqrt{\frac{1}{n}}$ for within-subject designs and $\sqrt{\frac{1}{n^{T}}+\frac{1}{n^{C}}}$ for between-subject designs to avoid the inherent dependence between *d*-values and their standard errors (Pustejovsky and Rodgers, 2019).

Analyses were conducted using R version 4.4.3 (R Core Team, 2025) with the help of the metafor (Viechtbauer, 2010) and clubSandwich (Pustejovsky, 2024) packages.

## Results

### Search results

**Figure 1.**
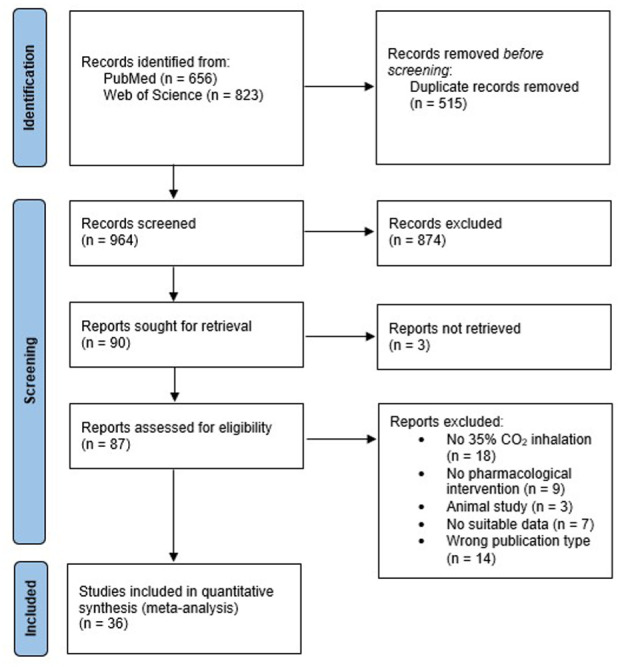
PRISMA 2020 flow diagram Source: Page et al. (2021).

The search via the two databases yielded 1479 publications. After duplicates (*n* = 515) were removed, titles and abstracts of 964 studies were screened. In the next step, inclusion and exclusion criteria were applied to the full text of the remaining 87 articles. In the end, 36 studies were selected for the quantitative meta-analysis. The PRISMA flow diagram (Figure 1) displays the search process.

### Characteristics of meta-analysed studies

Table 1 summarizes the characteristics of the included studies. The oldest study was published in 1984 (Van den Hout and Griez, 1984) and the most recent one in 2024 (de Vos et al., 2024).

**Table 1.** Key characteristics of studies included in the meta-analysis.

Author (year)	Number of participants	Population	Mean age (SD)	Sex (% female)	Pharmacological intervention(s)	Main mode of action^a^	Dosage	Study aim	Extracted outcomes
Bailey et al. (2009)	12	HC	21	50%	Alprazolam	Benzodiazepine receptor agonist	1 mg, single dose	↓	VAS-A; PSI
					Zolpidem	PAM	5 mg, single dose	↓	VAS-A; PSI
Battaglia et al. (2001)	12	PD^b^	27.4 (5.1)	50%	Pirenzepine	Muscarinic M1 receptor antagonist	100 mg, single dose	↓	VAS-A; PSL
					Biperiden Hydrochloride	Muscarinic receptor antagonist (non-selective)	100 mg, single dose	↓	VAS-A; PSL
Ben-Zion et al. (1999)	14	HC			Metergoline	Serotonin receptor antagonist	4 mg, single dose	↑	Anxiety Subscale of NIMH Self-Rating Scale
Bertani et al. (2001)	15	PD^c^	25.9 (8.7)	73%	Citalopram	Serotonin reuptake inhibitor	10 mg/day × 1 week	↓	VAS-A
Colasanti et al. (2011)	18	HC	25 (5.5)	44%	Tryptophan depletion	Serotonin synthesis inhibitor	Once	↑	VAAS; PSL
					Tryptophan	Serotonin precursor	5.15 g, single dose	↓	VAAS; PSL
Coryell and Rickels (2009)	32	High risk of PD^b^	24.4 (3.6)	72%	Escitalopram	Serotonin reuptake inhibitor	10 mg/day × 2 weeks	↓	VAS-A
Cosci et al. (2004)	8	HC	36.6	38%	Alcohol	GABA-A receptor modulator	20 g (female) or 28 g (male), single dose	↓	VAS-A; PSL
Cosci et al. (2005)	10	PD^b^	42.1	50%	Alcohol	GABA-A receptor modulator	18.9 g (female) or 26.09 g (male), single dose	↓	VAS-A; PSL
	16	HC	39	44%	Alcohol	GABA-A receptor modulator	18.9 g (female) or 26.09 g (male), single dose	↓	VAS-A; PSL
Cosci et al. (2006)	33	HC	26.3 (9.8)	79%	Nicotine	Nicotinic acetylcholine receptor agonist	10 mg patch, single dose	↑	VAS-A; PSL
De Cort et al. (2017)	44	HC	27.9 (10.5)	61%	Pentagastrine	Gastrin receptor agonist	2 mcg/kg, single dose	↑	PSL
De Vos et al. (2024)	34	HC	21.9 (5.9)	68%	Atenolol	β1-adrenergic receptor antagonist	100 mg, single dose	↓	VAS-F; PSL
					Metoprolol	β1-adrenergic receptor antagonist	100 mg, single dose	↓	VAS-F; PSL
Diaper et al. (2013)	54	HC	23.1 (4.68)	46%	Venlafaxine	Serotonin reuptake inhibitor	75 mg/day × 3 days 112.5 mg/day × 4 days 150 mg/day × 14 days	↓	VAS-A; PSI
					Pregabalin	Glutamate channel blocker	100 mg/day × 7 days 200 mg/day × 14 days	↓	VAS-A; PSI
Esquivel et al. (2009)	24	HC	24.0 (4.5)	67%	Naltrexone	Opioid antagonist	50 mg, single dose	↑	VAS-A; PSL
Hood et al. (2006)	14	HC	34.5	64%	Tryptophan depletion	Serotonin synthesis inhibitor	Once	↑	VAS-A; PSI
Kaufmann et al. (2021)	30	HC		30%	ACT-539313	selective orexin-1 receptor antagonist	200 mg/day × 3 days	↓	PSI
Klaassen et al. (1998)	15	HC	29 (4)	0%	Tryptophan depletion	Serotonin synthesis inhibitor	Once	↑	VAS-A; PSL-IIIR
Kushner et al. (1996)	31	PD^b^	31 (8)	71%	Alcohol	GABA-A receptor modulator	0.085 g/dL BAL, single dose	↓	State Anxiety; API
Lehman et al. (2002)	60	PD^d^	35.5 (12.0)	78%	Alcohol	GABA-A receptor modulator	0.06 g/dL BAL, single dose	↓	Anxiety rating
Meiri et al. (2001)	14	HC			Metergoline	Serotonin receptor antagonist	4 mg, single dose	↑	VAS-A
Nardi et al. (1999)	6	PD^d^	33.5 (7.2)	83%	Clonazepam	GABA – PAM	2 mg/day × 10 days	↓	SUDS
Nardi et al. (2000)	22	PD^d^	37.5 (11.2)	59%	Clonazepam	GABA – PAM	2 mg, single dose	↓	SUDS
Papadopoulos et al. (2010)	12	HC	26	33%	Hydroxyzine	Histamine antagonist	25 mg, single dose	↓	VAS-A; PSI
					Propranolol	Non-selective β-adrenergic receptor antagonist	40 mg, single dose	↓	VAS-A; PSI
					Flupentixol	Dopamine antagonist	0.5 mg, single dose	↓	VAS-A; PSI
Perna et al. (1994)	18	PD^c^	39.6 (10.1)		Toloxatone	Reversible MAO-A inhibitor	200–600 mg/day × 3 days 600 mg/day × 4 days	↓	VAS-A
Perna et al. (1997)	39	PD^c^	34.3 (9.7)		Clomipramine	Serotonin reuptake inhibitor	10 mg/day × 4 days 20 mg/day × 4 days	↓	VAS-A
					Fluvoxamine	Serotonin reuptake inhibitor	50 mg/day × 8 days	↓	VAS-A
Perna et al. (2002)	108	PD^c^	32.3 (9.2)	48% Female	Imipramine	Serotonin reuptake inhibitor	10 mg/day × 3 days 25 mg/day × 4 days 50 mg/day × 7 days 75 mg/day × 16 days	↓	VAS-A
					Clomipramine	Serotonin reuptake inhibitor	10 mg/day × 3 days 25 mg/day × 4 days 50 mg/day × 7 days 75 mg/day × 16 days	↓	VAS-A
					Paroxetine	Serotonin reuptake inhibitor	10 mg/day × 7 days 20 mg/day × 8 days 30 mg/day × 15 days	↓	VAS-A
					Sertraline	Serotonin reuptake inhibitor	25 mg/day × 7 days 50 mg/day × 8 days 75 mg/day × 15 days	↓	VAS-A
					Fluvoxamine	Serotonin reuptake inhibitor	50 mg/day × 7 days 100 mg/day × 8 days 150 mg/day × 15 days	↓	VAS-A
Perna et al. (2004)	14	PD^c^	32.4 (10.9)	64%	Paroxetine	Serotonin reuptake inhibitor	10 mg/day × 1 week	↓	VAS-A
	14	PD^c^	34.3 (8.6)	43%	Reboxetine	Norepinephrine reuptake inhibitor	10 mg/day × 1 week	↓	VAS-A
Pols et al. (1994)	20	HC	24.1	55%	Yohimbine	α2-adrenergic receptor antagonist	20 mg, single dose	↑	VAS-A; PSL
Pols et al. (1996)	20	PD^c^	34.5 (8.8)	75%	Alprazolam	Benzodiazepine receptor agonist	1 mg, single dose	↓	VAS-A; PSL
Pols et al. (1996)	11	PD^d^	35.6 (10.9)	82%	Fluvoxamine	Serotonin reuptake inhibitor	50 mg/day × 4 days 100 mg/day × 4 days 150–200 mg/day × 36 days	↓	VAS-A; PSL
Pols et al. (1999)	27	HC			CCK-4	Cholecystokinin-B (CCK-B) receptor agonist	5 mcg, single dose	↑	VAS-A; PSL
Schoepp et al. (2003)	27	PD^c^			LY354740	Group II metabotropic glutamate receptor (mGluR2/3) agonist	200 mg/day × 4 weeks	↓	VAS-A
Schruers et al. (2000)	40	HC	32.1 (11.2)	65%	CCK-4	CCK-B receptor agonist	10 mcg, single dose	↑	VAS-A; PSL
Schruers et al. (2000)	24	PD^c^	40 (11.5)	63%	Tryptophan depletion	Serotonin synthesis inhibitor	Once	↑	VAS-A; PSL
Schruers et al. (2002)	24	PD^c^	39.9 (10.7)	46%	L-5-hydroxytryptophan	Serotonin (5-HT) biosynthetic precursor	200 mg, single dose	↓	VAS-A; PSL
	24	HC	29.8 (11.7)	58%	L-5-hydroxytryptophan	Serotonin (5-HT) biosynthetic precursor	200 mg, single dose	↓	VAS-A; PSL
Schruers and Griez (2004)	10	PD^d^	39 (11)	70%	Tianeptine	Unclear	37.5 mg/day × 6 weeks		VAS-A; PSL
	10	PD^d^	44 (11)	60%	Paroxetine	Serotonin reuptake inhibitor	20 mg/day × 1 week 40 mg/day × 5 weeks	↓	VAS-A; PSL
Van den Hout and Griez (1984)	20	HC		50%	Propranolol	Non-selective β-adrenergic receptor antagonist	60 mg, single dose	↓	PSL

PD: panic disorder; HC: healthy controls; VAS-A: visual analogue scale anxiety; PSI: panic symptom inventory; PSL: panic symptom list; VAAS: visual analogue scale for affect; API: acute panic inventory; SUDS: Subjective Units of Disturbance Scale; CCK-4: cholecystokinin-tetrapeptide; CCK-B: cholecystokinin-B.

a According to the mode of action as listed in the neuroscience-based nomenclature (Zohar et al., 2014) app, if available.

b PD without reporting agoraphobia status.

c PD with or without agoraphobia.

d PD with agoraphobia.

↑The aim or hypothesis of the study was to examine whether the respective pharmacological intervention could enhance the effect of the 35% CO_2_ inhalation.

↓The aim or hypothesis of the study was to examine whether the respective pharmacological intervention could inhibit the effect of the 35% CO_2_ inhalation.

### Meta-analysis

In total, 36 studies reported the effects of a pharmacological intervention on the subjective experience of the CO_2_ inhalation, for a total of 980 participants. Several studies examined multiple pharmacological interventions, resulting in a total of 48 effect sizes for the meta-analysis of the effects of the interventions on some measure of anxiety and 34 for the effects on PA symptoms. Analyses were run separately for these two different types of outcomes.

### Inhibiting the response to 35% CO_2_

#### Self-reported anxiety

**Figure 2.**
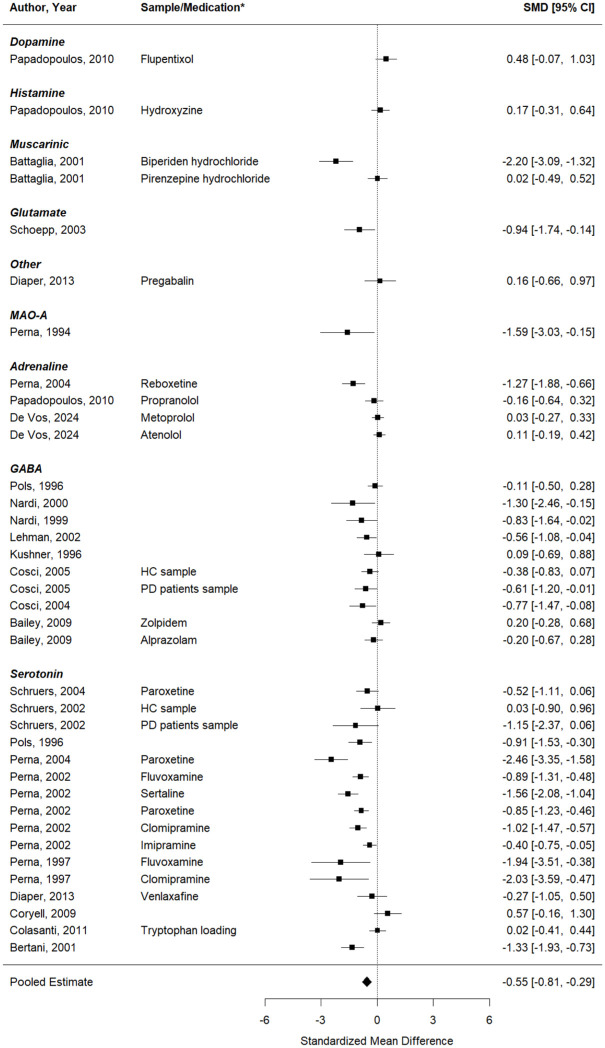
Forest plot of the meta-analysis of studies that aimed to test whether the respective pharmacological intervention could inhibit the effect of the CO2 inhalation on self-reported anxiety. *If data from multiple samples or pharmacological interventions were extracted from the same paper, the key characteristic of each entry is provided in the column “Sample/Medication” to explain how this entry differs from the other entry of the same paper (e.g. two different pharmacological interventions were tested, or the same intervention was tested in two different samples).

First, results were computed for studies that aimed to test whether the respective pharmacological intervention could inhibit the effect of the CO_2_ inhalation on self-reported anxiety (23 studies, 37 effect sizes). The effect sizes of the individual studies and the results of the meta-analysis are depicted in a forest plot in Figure 2. A significant decrease in induced anxiety was seen after a pharmacological intervention, compared to inhalations performed with a placebo or at baseline. The pooled effect was − 0.55 (95%CI: −0.81 to −0.29), *p* < 0.0001, but considerable heterogeneity was present in the effects (*Q* (df = 36) = 173.78, *p* < 0.0001; $I_{\mathrm{study}}^{2}$ = 38.8%, $I_{\mathrm{effect}}^{2}$ = 45.8%, PI: −1.76 to 0.65).

Second, a meta-regression model with medication target (with the levels: serotonergic, the GABAergic, or another system) as moderator was tested. No significant differences in the effects on self-reported anxiety were found between the pharmacological interventions targeting the serotonergic or GABAergic system, compared to other pharmacological targets (QM (df = 2) = 1.96, *p* = 0.38).

#### Self-reported PA symptoms

**Figure 3.**
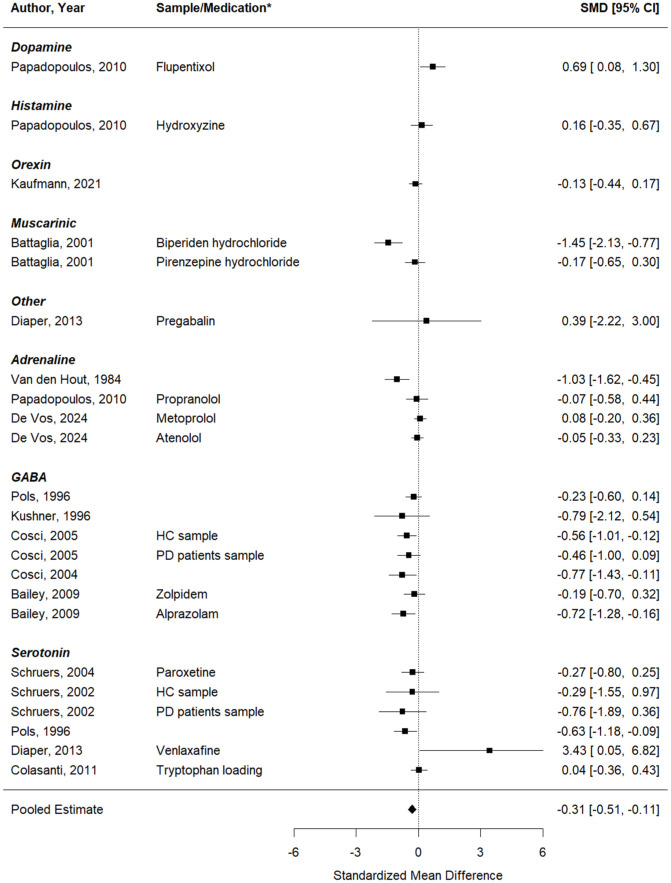
Forest plot of the meta-analysis of studies that aimed to test whether the respective pharmacological intervention could inhibit the effect of the CO_2_ inhalation on self-reported panic symptoms. *If data from multiple samples or pharmacological interventions were extracted from the same paper, the key characteristic of each entry is provided in the column “Sample/Medication” to explain how this entry differs from the other entry of the same paper (e.g. two different pharmacological interventions were tested, or the same intervention was tested in two different samples).

The same analysis was performed as above, now looking at the effects on self-reported PA symptoms (15 studies, 23 effect sizes). Similarly, a significant decrease in induced PA symptoms was seen after a pharmacological intervention, compared to inhalations performed under a placebo or at baseline (Figure 3). The pooled effect was −0.31 (95%CI: −0.51 to −0.11), *p* = .0026 (*Q*(df = 22) = 59.75, *p* < 0.0001; $I_{\mathrm{study}}^{2}$ = 11.3%, $I_{\mathrm{effect}}^{2}$ = 57.3%, PI: −1.07 to 0.36). Based on the meta-regression model with medication target as moderator, no significant differences in the effects on self-reported PA symptoms were found between the medication targets (QM (df = 2) = −1.01, *p* = 0.39).

### Enhancing the response to 35% CO_2_

#### Self-reported anxiety

**Figure 4.**
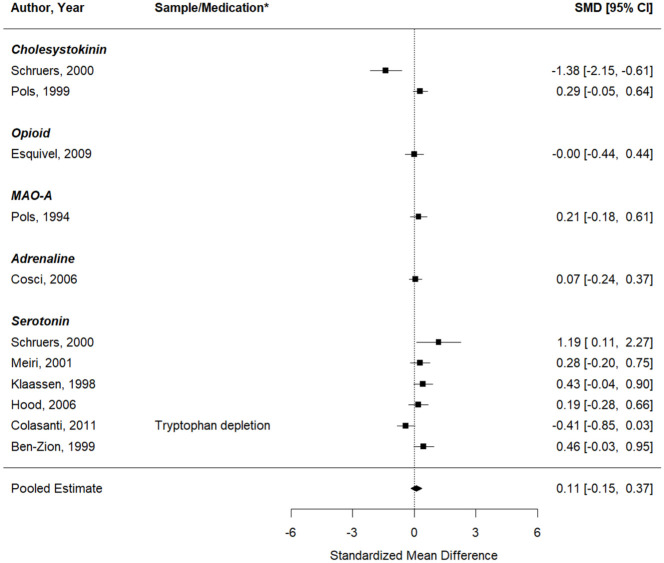
Forest plot of the meta-analysis of studies that aimed to test whether the respective pharmacological intervention could enhance the effect of the CO_2_ inhalation on self-reported anxiety. *If data from multiple samples or pharmacological interventions were extracted from the same paper, the key characteristic of each entry is provided in the column “Sample/Medication” to explain how this entry differs from the other entry of the same paper (e.g. two different pharmacological interventions were tested, or the same intervention was tested in two different samples).

First, results were computed for studies that aimed to test whether the respective pharmacological intervention could enhance the effect of the CO_2_ inhalation on self-reported anxiety (11 studies, 11 effect sizes). The results are depicted in a forest plot in Figure 4. There was no significant change in anxiety in the studies that aimed to increase the response to the CO_2_ inhalation (pooled effect = 0.11 (95%CI: −0.15 to 0.37), *p* = 0.41; *Q* (df = 10) = 29.59, *p* = 0.001; $I^{2}$ = 71.4%, PI: −0.65 to 0.87). The meta-regression model including medication target (with only the levels serotonergic versus other, since medications targeting the GABAergic were always used with the aim to decrease the effect of the inhalation) did not indicate any significant differences (QM (df = 1) = 1.35, *p* = 0.24).

#### Self-reported PA symptoms

**Figure 5.**
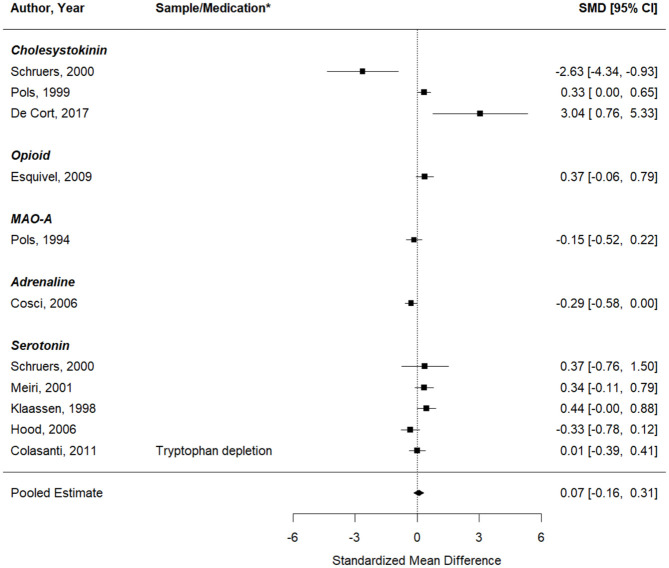
Forest plot of the meta-analysis of studies that aimed to test whether the respective pharmacological intervention could enhance the effect of the CO_2_ inhalation on self-reported panic symptoms. *If data from multiple samples or pharmacological interventions were extracted from the same paper, the key characteristic of each entry is provided in the column “Sample/Medication” to explain how this entry differs from the other entry of the same paper (e.g. two different pharmacological interventions were tested, or the same intervention was tested in two different samples).

Second, the same analysis was performed, now looking at the effects on self-reported PA symptoms (11 studies, 11 effect sizes). Similarly, no significant change in induced PA symptoms was seen after a pharmacological intervention, compared to inhalations performed under a placebo or at baseline (Figure 5). The pooled effect was 0.07 (95%CI: −0.16 to 0.31), *p* = 0.54 (*Q* (df = 10) = 35.02, *p* = 0.0001; $I^{2}$ = 61.0%, PI: −0.54 to 0.68). No significant differences depending on the medication target were found (QM (df = 1) = 0.20, *p* = 0.66).

### Increased versus decreased availability of serotonin

#### Self-reported anxiety

A further meta-regression was performed for the studies primarily targeting serotonin. The number of studies allowed to test whether there is a different effect of pharmacological interventions aiming to enhance or inhibit the effect of serotonin signalling in the brain. This was a significant moderator (QM (df = 1) = 12.74, *p* = 0.0004). More specifically, an increase in the availability of serotonin is related to a significant reduction of the response to the CO_2_ inhalation (pooled effect = −.81 (95%CI: −1.13 to −.48), *p* < 0.0001), compared to a control condition. There was no significant change in anxiety after the use of a pharmacological intervention that decreased the availability of serotonin (pooled effect = .28 (95%CI: −.22 to 0.78, *p* = 0.27).

#### Self-reported PA symptoms

The same meta-regression was performed to test whether there is a different effect on self-reported PA symptoms of pharmacological interventions aiming to increase versus decrease the availability of serotonin. However, in this case, this was not a significant moderator (QM (df = 1) = 2.44, *p* = 0.12).

### Sensitivity analyses and publication bias

#### Self-reported anxiety

The standardized residuals for the model testing the effects of medications that aimed/hypothesized to inhibit the anxiety response yielded two potential outliers (Supplemental Figure 1a). Using Cook’s distance, one influential study was also identified (Supplemental Figure 1b). Excluding these potential outliers and influential studies from the model did not yield considerably different results (with all effect sizes: pooled effect = −0.55 (95%CI: −0.81 to −.29), *p* <0.0001; without: pooled effect = −0.45 (95%CI: −0.68 to −0.22), *p* = 0.0001). No evidence for a publication bias came forward from the funnel plot (Supplemental Figure 1c) and the regression test for asymmetry in the funnel plot (*p* = 0.35).

#### Self-reported PA symptoms

The standardized residuals for the model testing the effects of medications that aimed/hypothesized to inhibit the panic response yielded one potential outlier (Supplemental Figure 2a). Using Cook’s distance, two influential studies were identified (Supplemental Figure 2b). Excluding these potential outliers and influential studies from the model did not yield considerably different results (with all effect sizes: −.31 (95%CI: −.51 to −.11), *p* = 0.0026; without: pooled effect = −.39 (95%CI: −.58 to −.20), *p* < 0.0001). No evidence for a publication bias came forward from the funnel plot (Supplemental Figure 2c) and the regression test for asymmetry in the funnel plot (*p* = 0.12).

## Discussion

The current meta-analysis showed that overall, studies successfully inhibited the response to the 35% CO_2_ inhalation with the use of pharmacological interventions, both in self-reported anxiety levels and in self-reported PA symptoms. Overall, studies did not succeed in enhancing the effects of the inhalation. Eleven different biological systems were targeted in the included studies; however, the majority of tested pharmacological interventions (mainly) targeted the serotonergic or GABAergic system. There were no differences in the induced effects between pharmacological interventions that target the serotonergic or GABAergic, or a different (main) biological system.

The meta-analysis of the studies that aimed or hypothesized to inhibit the induced panic response comprises studies with different samples and pharmacological interventions. Even though heterogeneous samples (PD patients with or without agoraphobia and HCs) and mixed methods (e.g. variable dosages of medication) were included in the analysis, we found an overall achieved inhibition of the experimentally induced panic response. Finding this overall decrease despite this heterogeneity supports the robustness of the CO_2_ model when studying potential inhibitors of panic.

Additionally, to studying inhibitors of the panic response, studying which pharmacological interventions can enhance the effects of the CO_2_ inhalation can provide further insights into which biological systems play a role in the panic response. However, our meta-analysis revealed that no significant increase in self-reported anxiety levels or PA symptoms was achieved. This can potentially be explained by the already intense nature of the induced panic response. The 35% CO_2_ inhalation has been shown to induce similar symptoms and sensations as naturally occurring PAs (Griez et al., 2007; Schruers et al., 2011), which could already be a ceiling effect, leaving no room for a magnification of this response. In order to study potential enhancers of the panic response, future studies can consider using a lower dosage of CO_2_. Increasing concentrations of CO_2_ have been shown to induce a dose-dependent increase in self-reported measures (Leibold et al., 2013).

Although an overall decrease in the response to the CO_2_ inhalation was found, including the (main) targeted biological system as a moderator did not show different effects of the distinct target systems (serotonergic or GABAergic system). In other words, according to the current results serotonergic and GABAergic drugs were evenly effective. Meta-analyses comparing the effects of different pharmacological treatments in PD identify the highest remission rates for SSRIs and benzodiazepines, compared to other pharmacological treatments (Bighelli et al., 2016; Chawla et al., 2022). Another recent meta-analysis found little differences in effectiveness (Guaiana et al., 2023). In the present meta-analysis, a specific robust differential effect for SSRIs and benzodiazepines in reducing experimentally induced PA symptoms and anxiety levels compared to other pharmacological treatments could not be found. However, if this distinction could be replicated in the 35% CO_2_ model in the future, it would provide additional strong support for the usefulness of the 35% CO_2_ model as a screening tool for the therapeutical effects of pharmacological interventions on PAs.

The number of studies only allowed testing whether there is a different effect of pharmacological interventions aiming to increase versus decrease the availability of serotonin (and not of GABA or other targets). An increase in the availability of serotonin led to a reduced anxiety response to the CO_2_ inhalation. This is in line with the suggested role of the serotonergic system in detecting changes in arterial CO_2_ levels (Corcoran et al., 2009; Richerson, 2004) and the triggered affective response (Richerson, 2004). The current results therefore provide further confirmation of the involvement of the serotonergic system in the modulation of PAs. In clinical practice, medications that enhance the availability of serotonin in the synaptic cleft are used as a first-line treatment for PD (Zulfarina et al., 2019). As evidenced by this meta-analysis, increased serotonin levels are associated with the same anxiety-reducing effect on the experimentally induced PAs by the CO_2_ model. Taken together, these results support the predictive value of pharmacological effect on the CO_2_-induced panic response for the clinical effects of medication, thus, supporting the usefulness of the CO_2_ model as a screening tool of the effects of pharmacological interventions on PAs.

The absence of an increase in self-reported anxiety levels and PA symptoms when the availability of serotonin is decreased by a pharmacological intervention is in line with the above hypothesized ceiling effect of the 35% CO_2_ inhalation by itself.

Overall, in this meta-analysis, we provided a quantitative summary of 35 studies with a total of 946 participants. We were able to provide an indication of the achieved modulations of the experimentally induced panic response to the 35% CO_2_ inhalation under the influence of different pharmacological manipulations. When setting the inclusion criteria, several choices were made that could limit the current results. Firstly, only studies employing the 35% CO_2_ model as PA induction were included. Including other PA induction methods, for example, with cholecystokinin-tetrapeptide (CCK-4; Bradwejn et al., 1990, 1991) could have resulted in more included studies and therefore an even more comprehensive overview. However, this was outside the scope of the current meta-analysis, in which we wanted to specifically look at the effects on the 35% CO_2_ method. Similarly, studies employing lower percentages of CO_2_ (for a prolonged period) were also not included in the present meta-analysis. For example, prolonged (20-minute) inhalation of 7.5% CO_2_ has also been found to induce physiological changes and to trigger anxiety up to a panic-like response. However, as the evoked symptoms resemble more some of the symptoms characterizing Generalized Anxiety Disorder, this inhalation paradigm is considered a model for Generalized Anxiety Disorder rather than for PD (Bailey et al., 2007). Secondly, in order to reach a large sample of studies, studies with different samples (HC and/or PD with or without agoraphobia) and different procedures (e.g. a single dose of the pharmacological manipulation vs. 3 weeks of a daily dose) were included in the meta-analysis. In HCs, a double inhalation of 35% CO_2_ is considered to have similar effects as a single vital capacity breath of 35% CO_2_ in PD patients (Schruers et al., 2011). In the current meta-analysis, both studies applying a single and double inhalation were used in the HC samples, and therefore, some studies might have used a weaker PA induction method compared to other studies. Nevertheless, it is expected that if these studies would have used a stronger PA induction method (double instead of single breath of CO_2_ in HCs), the achieved inhibitory effects of the pharmacological interventions would have been even stronger.

Future studies assessing the effects of pharmacological interventions targeting different biological systems than the serotonin system are needed to perform a proper comparison between the effectiveness of different targets in reducing the experimentally induced panic response. Moreover, to fully validate the usefulness of the CO_2_ model as a screening tool for clinically relevant pharmacological interventions, a meta-regression is needed comparing the effects of effective versus ineffective interventions in the clinic, which was not yet possible with the current set of studies. Furthermore, in recent years, more studies have employed psychophysiological measures of the panic response next to self-report measures (Blechert et al., 2010; Bystritsky et al., 2000; Leibold et al., 2013). A meaningful quantification of those results could not be made yet. However, if future studies keep including those psychophysiological outcomes, the effects of pharmacological interventions on these can be considered and compared with the effects on the self-report measures with a meta-analysis.

In conclusion, our systematic search and following meta-analysis provide further establishment of the usefulness of the 35% CO_2_ model in the screening of the effects of pharmacological interventions on PAs. Specifically, for interventions that are aimed/hypothesized to have a PA-inhibiting effect, and thus have potential therapeutic benefits. In line with meta-analyses comprising studies assessing the effects of pharmacological treatments in PD, the majority of studies addressed the PA-reducing effects of interventions that increase the availability of serotonin in the synaptic cleft. In sum, based on the results, we advocate the use of the 35% CO_2_ model in panic-related drug research. First, as we can induce panic in healthy volunteers, the model has value in assessing drug effects on panic for more basic research questions. Second, testing drug effects on the CO_2_ challenge in healthy volunteers provides a screening step for potential new drugs in the treatment of panic. Screening this first in healthy volunteers offers the opportunity to assess the drug effects in a rather homogeneous sample and without the need to put a burden on patients with drugs that appear to be ineffective in decreasing panic responses. Additionally, we highly recommend the use of the challenge in PD patients. Testing the effects of drugs on patients’ responses to the challenge offers the opportunity to screen the drug effects on experimental panic (as naturally occurring PAs happen irregularly) and in a controlled setting, before engaging patients in larger clinical trials where measures like symptom reductions and remission rates can be taken as an outcome of drug effects.

## Supplemental Material

sj-docx-1-jop-10.1177_02698811251378756 – Supplemental material for Pharmacological effects on 35% CO2 panic induction: A meta-analysis Supplemental material, sj-docx-1-jop-10.1177_02698811251378756 for Pharmacological effects on 35% CO2 panic induction: A meta-analysis by Jette H. de Vos, Alissa Haj Yahya, Wolfgang Viechtbauer, David E. J. Linden, Koen R. J. Schruers and Nicole K. Leibold in Journal of Psychopharmacology
