# Supplementary material for: Pharmacological effects on 35% CO2 panic induction: A meta-analysis
Source: J Psychopharmacol. 2025 Oct 29;39(12):1397–408. doi: 10.1177/02698811251378756 (PMC12672939; doi:10.1177/02698811251378756)
Supplement: sj-docx-1-jop-10.1177_02698811251378756 – Supplemental material for Pharmacological effects on 35% CO2 panic induction: A meta-analysis [file sj-docx-1-jop-10.1177_02698811251378756.docx]

**Methods**

Pubmed search term:

("Panic disorder"[MeSH Terms] OR "Panic"[MeSH Terms] OR "Panic"[Title/Abstract]) AND ("carbon dioxide"[MeSH Terms] OR ("CO2"[Title/Abstract] OR "co 2"[Title/Abstract] OR "carbon dioxide"[Title/Abstract]))

Web of Science search term:

https://www.webofscience.com/wos/woscc/summary/a0be5a9a-ae48-41d9-ab47-dca208c10077-7c992c21/relevance/1

**Results**

Sensitivity analyses and publication bias

Self-reported anxiety


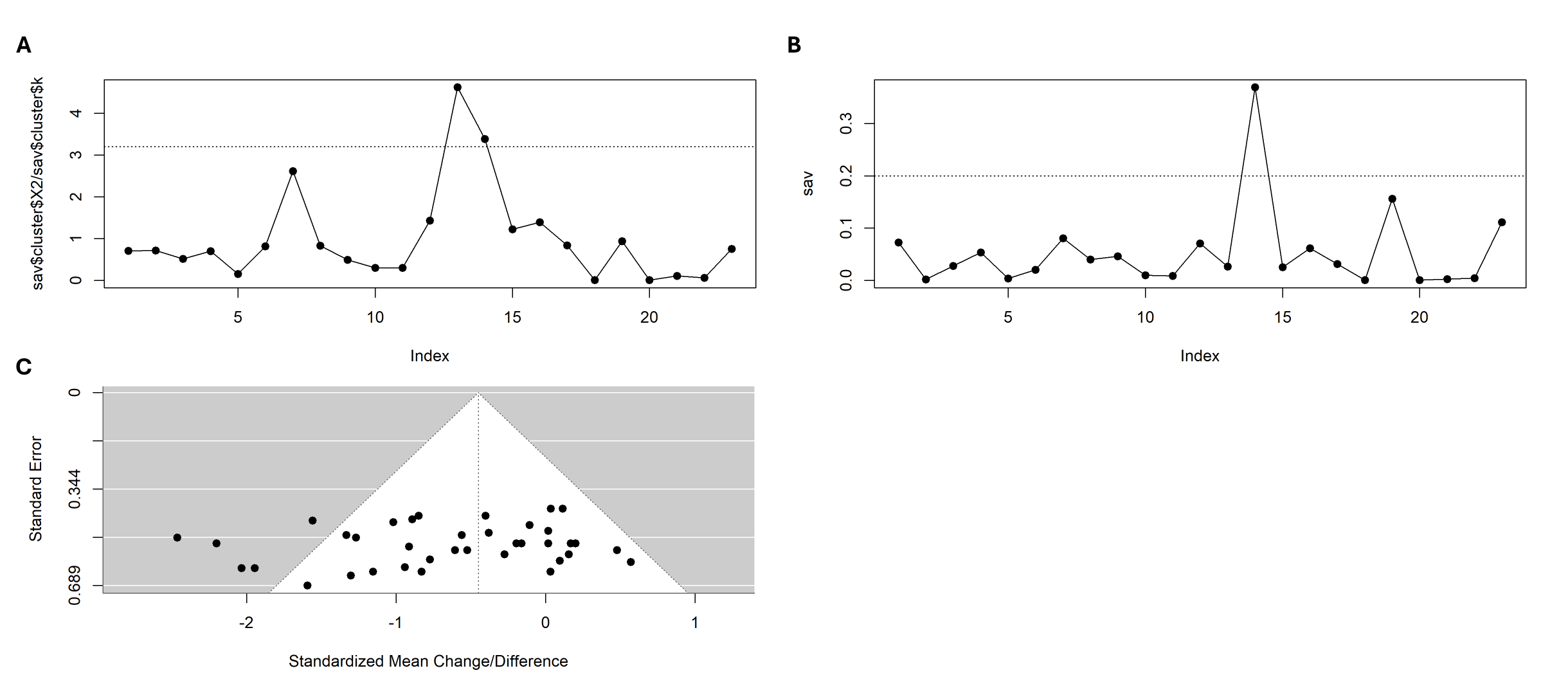


**Figure S1.** Plots illustrating the performed sensitivity analyses for the model testing the effects of the entries aiming/hypothesizing to inhibit the self-reported anxiety after experimental panic induction. (A) standardized residuals are represented on the y-axis and the individual entries are on the x-axis. The dotted line indicates the cut-off used for outliers. (B) Cook’s distances are represented on the y-axis and the individual entries are on the x-axis. The dotted line indicates the cut-off used for influential entries. (C) Funnel plot

Self-reported panic symptoms


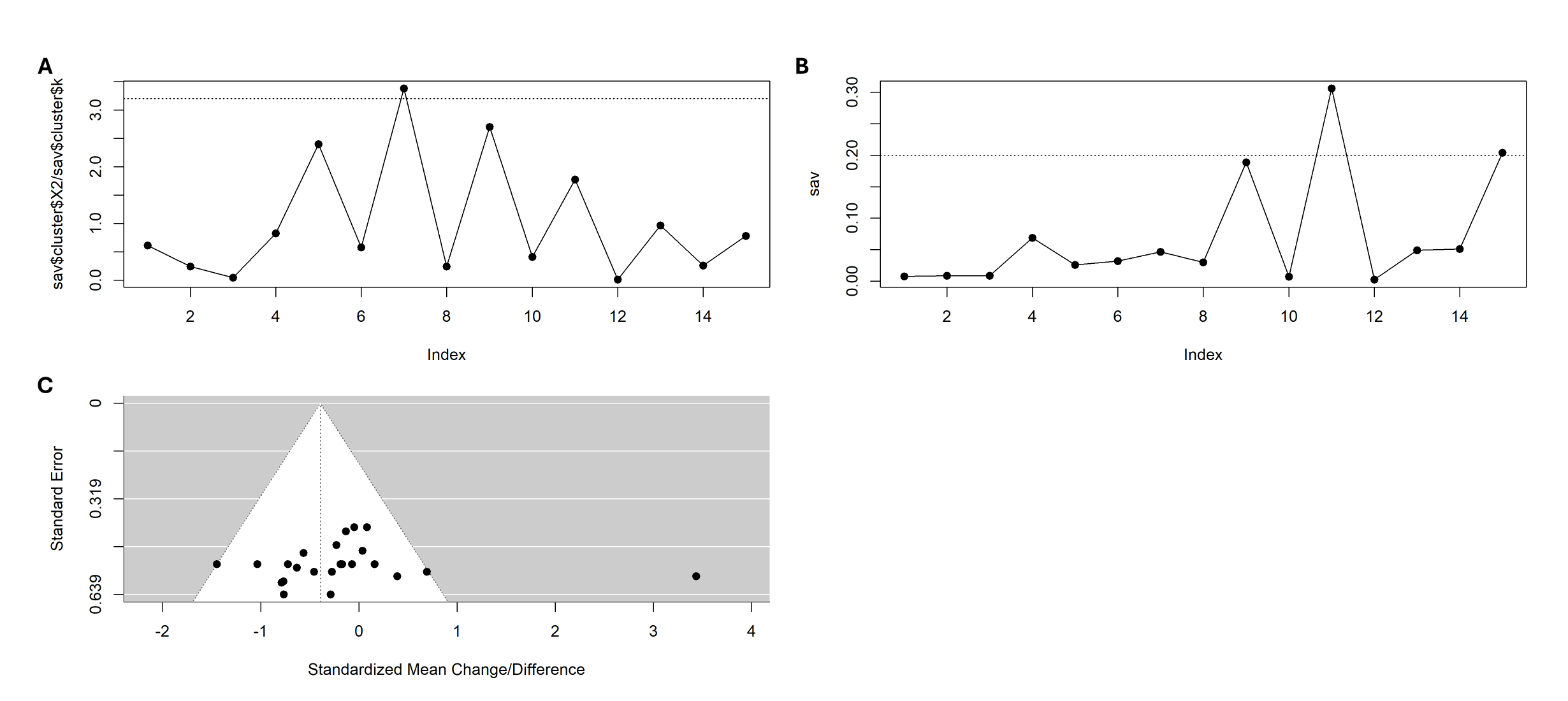


**Figure S2.** Plots illustrating the performed sensitivity analyses for the model testing the effects of the entries aiming/hypothesizing to inhibit the self-reported panic symptoms after experimental panic induction. (A) standardized residuals are represented on the y-axis and the individual entries are on the x-axis. The dotted line indicates the cut-off used for outliers. (B) Cook’s distances are represented on the y-axis and the individual entries are on the x-axis. The dotted line indicates the cut-off used for influential entries. (C) Funnel plot
